# Correlation among sleep quality, physical frailty and cognitive function of the older adults in China: the mediating role

**DOI:** 10.3389/fpubh.2023.1143033

**Published:** 2023-08-23

**Authors:** Shuzhi Peng, Yanping Chen, Jie Li, Yan Wang, Xingyue Liu, Ying Wang, Sainan Gu, Mengyun Pei, Peng Zhang

**Affiliations:** ^1^Graduate School of Nursing, Shanghai University of Medicine and Health Sciences, Shanghai, China; ^2^Graduate School of Nursing, Shanghai University of Traditional Chinese Medicine, Shanghai, China; ^3^Department of General Surgery, Funing People’s Hospital, Yancheng, Jiangsu, China; ^4^School of Nursing, Xuzhou Medical University, Xuzhou, Jiangsu, China; ^5^School of Public Health, Anhui Medical University, Hefei, Anhui, China; ^6^Graduate School of Nursing, Huzhou University, Huzhou, Zhejiang, China; ^7^School of Management, Hainan Medical University, Haikou, Hainan, China; ^8^School of Clinical Medicine, Shanghai University of Medicine and Health Sciences, Shanghai, China

**Keywords:** community, the older adults, physical frailty, sleep quality, cognitive function

## Abstract

**Objective:**

To explore the correlation among sleep quality, physical frailty, and cognitive function in the older adults in community, and to explore the mediating role of sleep quality.

**Methods:**

A total of 1,182 community-based older adults were investigated with frailty phenotype (FP), Pittsburgh sleep quality index (PISQI), Montreal cognitive assessment (MoCA) and self-made general information questionnaire.

**Results:**

The incidence of physical frailty among the older adults in the community was 25.8% and the incidence of cognitive decline was 19.5%. Cognitive function was negatively correlated with physical frailty (*r* = −0.236, *p* < 0.01) and sleep quality (*r* = −0.558, *p* < 0.01). Sleep quality was positively correlated with physical frailty (*r* = 0.337, *p* < 0.01).

**Conclusion:**

The physical frailty of the older adults has a direct prediction effect on cognitive function, and is regulated by the mediating role of sleep quality. Sleep quality partially mediates the relationship between cognitive dysfunction and physical frailty, which is a new insight into the study of cognition and physical frailty in the older adults. In the future, we can take measures to improve the sleep quality of the older adults, so as to reduce the occurrence of cognitive dysfunction and physical frailty of the older adults.

## 1. Introduction

Auyeung et al. study found physical frailty was associated with cognitive decline over four years period (1). Physical frailty is a state of increased vulnerability and reduced responsiveness to stressors, which can lead to fall, hospitalization, disability, and mortality (2). Cognitive function is a process in which individuals actively understand the world through psychological activities after contact with the outside world, including memory and delayed memory, attention, language, execution and other fields. When damage occurs in one or more fields, it is cognitive dysfunction, which will affect the social function and quality of life of individuals to different degrees. In severe cases, it may even lead to death (3). Changes in cognitive function are associated with physical frailty, with the growth of age, the older adults will inevitably face a series of decline and loss of body function (4). Therefore, the prevention and early treatment of cognitive decline and physical frailty have important scientific and social significance.

The research by Brigola et al. (5) showed that physical frailty was a strong risk factor for cognitive decline in the older adults. The cognitive ability of the frailty older adults was lower than that of the non-frailty older adults. Canevelli et al. (6) found in their research that decreased cognitive function also increases the risk of physical frailty, and older adults with decreased cognitive function or dementia are more likely to be frailty. Physical frailty and cognitive decline in the same older adults often occur together, and their incidence increases with age (2). Studies have shown that cognitive decline can be predicted by physical frailty, and the risk of cognitive decline in the older adults with physical frailty will be increased (7). Longitudinal studies abroad have found that the cognitive function of the older adults with physical frailty declines faster than that of the non-physical frailty older adults. However, the underlying mechanism of the relationship between physical frailty and cognitive function remains unclear.

One possible mechanism is somnipathy, which is a common physiological state in the older adults, which seriously affects the quality of life of the older adults and increases the incidence of physical frailty to a certain extent (8). In recent years, sleep disorder, as a risk factor for physical frailty, has gradually attracted extensive attention by researchers (9). According to statistics, the prevalence of sleep disorders in the older adults aged 60 and over is 42.3% (10). At the same time, the prevalence of sleep disorders in patients with cognitive decline is also high. Sleep disorders not only accelerate cognitive decline and lead to an increase in the mortality rate, but also increase the economic and psychological burden of caregivers (11).

Sleep disorders, physical frailty and cognitive decline are common among the older adults and are often overlooked. Although they are not directly life-threatening, their long-term existence can lead to an increased risk of dementia in the older adults, affect the quality of life of the older adults, and have an interaction with the health status of the older adults (12). At present, the relationship between sleep quality and cognitive function and physical frailty of the older adults is still unclear.

Most previous studies focused on the relationship between cognitive function and physical frailty, but few studies explored the potential mechanism of this relationship. In order to make up this gap, this study aims to explore the mediating role of sleep disorder in the relationship between cognitive function and physical frailty of the older adults in the Chinese community. To this end, we have the following specific goals. First, we will determine the prevalence of frailty and cognitive impairment among the older adults in the Chinese community. Secondly, we will study the relationship between sleep disorder, cognitive function and physical frailty of the older adults. Finally, we will verify the mediating role of sleep disorder between cognitive function and physical frailty.

## 2. Methodology and methods

### 2.1. Sampling method

In this study, stratified cluster sampling was used to investigate the older adults in the community in China. According to the economic development level of each district and county, we randomly divided three districts, five counties and one county-level city in Yancheng City, Jiangsu Province, China into three groups. Using random table method, each group selects one district and county, each district and county randomly selects two streets, and each street randomly selects two communities. A total of 12 communities were investigated, and the older adults who met the inclusion criteria in this community were investigated. The sample collection time of our study is from May 1st to June 1st, 2022.

### 2.2. Participants

Inclusion criteria: ① Age ≥ 60 years old; ②Informed consent and voluntary participation in research. Exclusion criteria: ① severe cognitive impairment; ② those with severe hearing and hearing impairment; ③ Those who have serious diseases and cannot cooperate. All participants obtained informed consent and signed the informed consent form. At the same time, this research was approved by the Ethics Review Committee of China Shanghai University of Medicine & Health Sciences (no. 2021-SMHC-01-015).

### 2.3. Survey method

We will take 12 community health service centers sampled by stratified cluster as survey sites, and each service center will set up an office as the survey point, and our investigators will conduct a unified questionnaire survey on the older adults in the community who meet the requirements. We have five investigators, including one attending doctor, two nurses and two graduate students, who passed the unified training before taking part in this study. The training content includes the definitions and standards of sleep, physical frailty and cognition, as well as matters needing attention in the process of sample collection. A total of 1,200 older adults in the community were investigated in this study. Among them, 18 older adults refused to cooperate with the investigation midway, and 1,182 older adults were eligible for the study.

### 2.4. Instruments and measurements

1. General information questionnaire: including gender, age, marital status, length of education, exercise, intellectual activity, social contact, fall history, and live in solitude, etc.
2. Montreal cognitive assessment (MoCA): this phenotype was prepared by Nasreddine et al. (13), in 2004 and included seven items, which were as follows: ① visuospatial skills; ② naming; ③ attention; ④ Language; ⑤ Abstraction; ⑥ Memory and delayed memory; ⑦ Calculation and orientation, the total score ranged from 0 to 30, and the higher the score, the better the cognitive function would be. A score of ≥26 indicates normal cognitive function and < 26 indicates cognitive decline. If the participant’s educational year is less than 12, the total score will be increased by 1 point.
3. Frailty phenotype (FP): FP was proposed by Freid et al. (14), and includes five phenotypes, as follows: ① unexplained weight loss; ② Self-incriminating fatigue; ③ Slow walking speed; ④ weak grip strength; (5) less physical activity. Total score of 0 ~ 5, the higher the score, the more serious the frailty. Score ≥ 3 is considered frailty, score of 1 ~ 2 is considered pre-frailty, 0 points are considered no frailty.
4. Pittsburgh sleep quality index (PSIQ): PSIQ compiled by Buysse et al. (15). The scale consists of 18 self-rated items and consists of 7 components, including ① subjective sleep quality; ② sleep latency; ③ sleep duration; ④ sleep efficiency; ⑤ sleep disorder; ⑥ use of hypnotic drugs; ⑦ daytime dysfunction, the scores of each component are 0 ~ 3, and the total score is 0 ~ 21. The higher the PSIQ score, the worse the sleep quality.

### 2.5. Statistical analysis

The database was established using EpiData 3.1 software. All data were entered and checked by two people. If there was any disagreement between the two people, the third person would check the data. SPSS 25.0 software was used for statistical analysis. The Kolmogorov–Smirnov test was used to determine the normality of continuous variables. Measurement data subject to normal distribution were described with mean ± standard deviation (SD), and t test was used for inter-group comparison. The non-normal distribution was described by the median and quartile, and Mann–Whitney U test was used for inter-group comparison, the correlation between variables was analyzed by Spearman’s correlation coefficient. Enumeration data were described by frequency and percentage, and inter-group comparison was performed by Chi-square test. PROCESS v4.1 was used to analyze the mediating role and the Bootstrap program in the software was used to test the mediating role of sleep quality in the relationship between physical frailty and cognitive function. A difference of *p* < 0.05 was statistically significant.

## 3. Results

The older adults in the community investigated in this study were aged 60–97, including 462 (39.09%) aged 60–70, 490 (41.46%) aged 70–80, 218 (18.44%) aged 80–90, and 12 (1.02%) aged over 90. Among them, 618 female (52.28%) and 184 unmarried (15.57%); 55 people (4.65%) have never received education. See Table 1 for other general information.

**Table 1:** General information of 1,182 participants [*n* (%), *M* (P25, P75)].

Variable	Frequency (%)
Age	73 (67.79)
Gender	
Female	618 (52.28)
Male	564 (47.72)
Marital status	
Married	666 (56.35)
Unmarried	184 (15.57)
Divorce	162 (13.71)
Widowed	170 (14.38)
Years of education	
0	55 (4.65)
1–6	357 (30.20)
6–12	649 (54.91)
>12	121 (10.24)
live in solitude	
Yes	686 (58.04)
No	496 (41.96)
Exercise	
Never	242 (20.47)
Average 1–3 times/week	387 (32.74)
Average 3–6 times/week	278 (23.52)
Every day	275 (23.27)
Intellectual activity	
Yes	687 (58.12)
No	495 (41.88)
History of falls	
Yes	531 (44.92)
No	651 (55.08)
Social contact	
Yes	815 (68.95)
No	367 (31.05)

The incidence rate of physical frailty, cognitive decline and sleep quality among the older adults in our survey is 30.37, 19.54, and 11.68%, respectively. According to Spearman’s correlation analysis, there is a significant negative correlation between MoCA score and FP score and PSQI score. There was a significant positive correlation between FP score and PSQI score. See Table 2 and Table 3 for details.

**Table 2:** Score of sleep quality and cognitive function and physical frailty.

Variable	*M* (P_25_,P_75_)	Score	Frequency	Percent (%)
MoCA score	27 (26,28)	26–30	951	80.46
		0–25	231	19.54
PSQI score	12 (9,14)	16–21	138	11.68
		11–15	586	49.58
		6–10	418	35.36
		0–5	40	3.38
FP score	2 (1,3)	3–5	359	30.37
		1–2	518	43.82
		0	305	25.80

**Table 3:** Correlation between sleep quality and cognitive function and physical frailty.

Variable	MoCA score	FP score	PSQI score
MoCA score	1		
FP score	−0.236**	1	
PSQI score	−0.558**	0.337**	1

The total scores of cognition function from 0 to 30. ≥26 indicates normal cognitive function, and < 26 indicates cognitive decline. The Chi-square test showed that cognitive function was not significant for Live in Solution (*p* > 0.05), but significant for Gender, Age, Marital status, Years of education, Exercise, Intellectual activity, social contact, history of falls, sleep quality and physical frailty (*p* < 0.05) (Table 4).

**Table 4:** Comparison of cognitive function with different characteristics.

Variable		Cognitive function (%)		χ^2^	*p*
		Decline	Normal		
Gender	Male	80 (34.63)	484 (50.89)	19.701	0.000**
	Female	151 (65.37)	467 (49.11)		
Age	60–70	69 (29.87)	393 (41.32)	31.635	0.000**
	70–80	106 (45.89)	384 (40.38)		
	80–90	47 (20.35)	171 (17.98)		
	>90	9 (3.90)	3 (0.32)		
Marital status	Married	81 (35.06)	585 (61.51)	59.566	0.000**
	Unmarried	62 (26.84)	122 (12.83)		
	Divorce	49 (21.21)	113 (11.88)		
	Widowed	39 (16.88)	131 (13.77)		
Years of education	0	42 (18.18)	13 (1.37)	188.947	0.000**
	1–6	108 (46.75)	249 (26.18)		
	6–12	81 (35.06)	568 (59.73)		
	>12	0 (0.00)	121 (12.72)		
Exercise	Never	98 (42.42)	144 (15.14)	128.193	0.000**
	1-3times/week	86 (37.23)	301 (31.65)		
	3-6times/week	5 (2.16)	273 (28.71)		
	Every day	42 (18.18)	233 (24.50)		
Intellectual activity	No	112 (48.48)	383 (40.27)	5.149	0.023*
	Yes	119 (51.52)	568 (59.73)		
Social contact	No	98 (42.42)	269 (28.29)	17.353	0.000**
	Yes	133 (57.58)	682 (71.71)		
History of falls	No	107 (46.32)	544(57.20)	8.896	0.003**
	Yes	124 (53.68)	407 (42.80)		
live in solitude	No	85 (36.80)	411 (43.22)	3.146	0.076
	Yes	146 (63.20)	540 (56.78)		
Sleep quality	Very good	0 (0.00)	40 (4.21)	343.430	0.000**
	Good	19 (8.23)	399 (41.96)		
	Not bad	108 (46.75)	478 (50.26)		
	Bad	104 (45.02)	34 (3.58)		
Physical frailty	No frailty	133 (57.58)	226 (23.76)	110.828	0.000**
	Pre-frailty	44 (19.05)	474 (49.84)		
	Frailty	54 (23.38)	251 (26.39)		

*p < 0.05; **p < 0.01.

**Figure 1:**
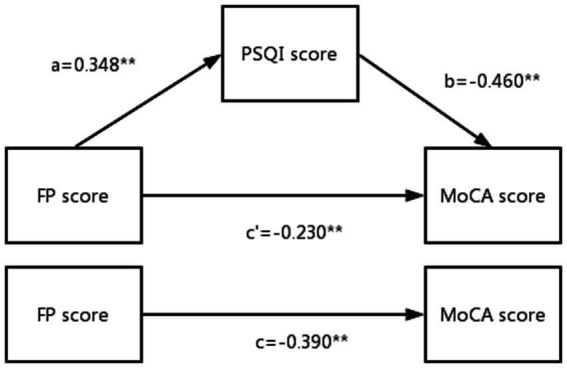
Mediating role test model diagram.

In the mediating role test, FP score has a significant influence relationship on MoCA score in Model 1 (*β* = −0.390, *p* < 0.01), indicating that the total effect is valid. In the test of model 2, FP score had a significant effect on PSQI score (*β* = 0.348, *p* < 0.01), while in the test of model 3, FP score had a significant effect on MoCA score (*β* = −0.230, *p* < 0.01), and sleep quality had a significant effect on cognitive function (*β* = −0.460, *p* < 0.01), therefore, the mediating role of sleep quality in the model is established, and it is part of the mediating role. See Table 5 and Figure 1 for details.

**Table 5:** Test results of mediating role by distribution regression method.

Model	Model 1		Model 2		Model 3	
Variable	MoCA score		PSQI score		MoCA score	
Notation	*β*	*t*	*β*	*t*	*β*	*t*
FP score	−0.390**	−14.558	0.348**	12.733	−0.230**	−9.120
PSQI score					−0.460**	−18.182
*R* ^2^	0.152		0.121		0.338	
Adjust *R*^2^	0.152		0.120		0.337	
*F*	211.945**		162.134**		300.870**	

* *p* < 0.05; ** *p* < 0.01.

The mediating role of health responsibility in the model is tested by Bootstarp technology, and it can be seen that the indirect effect value is −0.510, and the 95% confidence interval (−0.352, −0.234) does not contain 0, which means that the indirect effect is established, so the sleep quality plays a significant mediating role in the model, and the confidence interval of the direct effect test results is (−0.510, −0.329). According to the calculation results of effect ratio, it can be seen that the effect ratio of sleep quality is 72% (Table 6).

**Table 6:** Bootstrap test results.

Effect relation	Effect value	LLCI	ULCI	Proportion of effect
Total effect	−0.710	−0.806	−0.615	
Direct effect	−0.420	−0.510	−0.329	59%
Indirect effect	−0.510	−0.352	−0.234	72%

## 4. Discussion

### 4.1. Comparison of cognitive function with different characteristics

The MoCA score ≥ 26 indicates normal cognitive function, and score < 26 indicates cognitive decline. The chi-square test showed that cognitive function was not significant for live in solution (*p* > 0.05), which was different from the results in previous studies (16). Previous studies have considered that living in solution is an independent risk factor for the cognitive decline in the older adults, which may be related to the better autonomy and self-care ability of the older adults living in solution. However, different cognitive functions showed significant differences in gender, age, marital status, years of education, exercise, intellectual activity, social contact, history of falls, sleep quality, and physical frailty (*p* < 0.05). With the increase of age, the cognitive function of the older adults inevitably declines, while the younger the age, the less the accumulation of *β*-amyloid protein in the brain, and the better the cognitive function of the brain, which is similar to the research results of Bae (17). Wada found that the longer the education time, the greater the total brain capacity of patients with mild cognitive impairment (MCI) (18). Although the education level of the older adults cannot be changed, they can learn through the acquired continuing education. For example, the older adults can take part in community universities for the older adults for further study when their own financial ability permits. Dong found in their research that exercise such as light housework helps the older adults maintain healthy cognitive function, and exercise is a protective factor for cognitive decline (19). The older adults with cognitive decline can be allowed to take appropriate physical activities to maintain their cognitive function. Su found that a healthy lifestyle with a wide range of interests is a protective factor for cognitive dysfunction (20). Therefore, the community should build a healthy and beneficial environment, cultivate the interests and hobbies of the older adults, and encourage the older adults to do whatever intellectual and physical activities they can at home. For the older adults in the community, we can intervene in many aspects, such as exercising and cultivating hobbies, so as to prevent, detect and intervene cognitive decline early.

### 4.2. Status of sleep quality, physical frailty and cognitive function

A total of 1,182 older adults in the community were surveyed in this study. Pittsburgh’s sleep quality scores ranged from 0 to 21, and those with PSQI scores ranging from 16 to 21 were considered to have poor sleep quality. In this study, 138 older adults with poor sleep quality accounted for 11.68%, which was consistent with some domestic research results (21). Sleep disorders will not only reduce the quality of life of the older adults, but also lead to or complicate physical and mental diseases and increase the risk of death. Therefore, the sleep problems of the older adults should be throughout our community care process. The incidence of physical frailty in this survey is 30.37%. The proportion of female physical frailty is higher than that of male. The main reason may be the rapid loss of estrogen in postmenopausal women, which has a negative impact on muscle strength, neuromuscular function and posture stability, leading to the increased incidence of senile female physical frailty. Among the older adults investigated in this study, 231 people (19.56%) have cognitive decline, which is lower than 31.3% reported by some domestic research institutes (22), which may be related to the differences of research subjects. Studies have also shown that cognitive function is related to educational level (23, 24). The older adults in this study have a relatively high educational level, so the detection rate of cognitive function decline is relatively low.

### 4.3. Correlation among sleep quality, physical frailty and cognitive function

Studies have pointed out that sleep quality is closely related to physical frailty and cognitive decline (25). This study found that the rate of cognitive decline of the older adults with poor sleep quality is higher than that of those with good sleep quality, and the rate of physical frailty of the older adults with poor sleep quality is also higher than that of the older adults with good sleep quality. According to Spearman correlation analysis, the correlation coefficient between FP score and MoCA score is −0.236, *p* < 0.01, which indicates that there is a significant negative correlation between physical frailty and cognitive function, that is, physical frailty may increase the risk of cognitive function decline, which is consistent with previous research results (26). Physical frailty may lead to the limitation of the older adults’ activities and the reduction of social communication, which may easily lead to problems such as loneliness and social isolation, while the reduction of external stimulation will also increase the risk of cognitive decline or even damage of the older adults. In addition, although physical frailty is not a disease, it is often related to arthritis, stroke, chronic pulmonary edema and other diseases, and diseases may bring adverse effects to blood circulation and nervous system, thus inducing cognitive impairment (27). The correlation coefficient between PSQI score and MoCA score was −0.558, and *p* < 0.01, indicating that the worse the sleep quality was, the higher the risk of cognitive decline would be. Sleep itself is a modifiable lifestyle factor that can be used as a protective factor against cognitive decline. The correlation coefficient between PSQI score and FP score was 0.337, and *p* < 0.01, indicating that the higher PSQI score was, the higher the FP score was, namely, the poorer the sleep quality was, the higher the risk of physical frailty was. This result is consistent with that of Chinese scholar Liu et al. (28). Paying attention to the relationship between sleep disorder and physical frailty in the older adults can provide a theoretical basis for early identification of high-risk factors for physical frailty, early prevention of the occurrence and development of physical frailty, and early implementation of nursing intervention.

### 4.4. The mediating role of sleep quality in the physical frailty and cognitive decline

The results of this study show that sleep quality plays a partial mediating role in the relationship between the older adults’ physical frailty and cognitive function. In other words, physical frailty not only directly affects the cognitive function of the older adults, but also indirectly affects the cognitive function by changing the sleep quality. The sleep quality reflects the individual’s ability to adapt to the use of internal and external resources (29). When the older adults face physical, psychological and social frailty, their self-confidence and sense of control decline and they cannot effectively use internal and external resources (30). As a result, the quality of sleep is reduced, leading to an increased risk of cognitive decline. Sleep quality can improve individual autonomy in daily living ability, promote physical recovery, alleviate depression symptoms, maintain mental health, enhance happiness, and improve quality of life (31). When facing the physical frailty, the older adults need to constantly make adjustments from physical, psychological and social aspects to adapt (32). The older adults with poor sleep quality are more likely to have a sense of helplessness and thus affect cognitive function due to the lack of internal and external resources available (33). Conversely, the older adults with good sleep quality were more energetic and able to maintain better cognitive function. It could be seen that sleep quality played an important buffer role between the physical frailty and cognitive function in the older adults.

### 4.5. Limitations

This study is a cross-sectional study, and the survey results can only represent the state of the respondents at that time. It is suggested that in the future, a large sample size, multi-center research on the cognitive function of the older adults in the community should be carried out, and longitudinal follow-up should be carried out to compare the differences of cognitive function with time.

## 5. Conclusion

The sleep quality of the older adults can be improved through health knowledge promotion, exercise, nutrition intervention, and group psychological counseling. Good sleep quality can enhance their resilience and strength to deal with adverse environment or negative events, thereby improving or even reversing physical frailty state of the older adults and avoiding the decline of their cognitive function.

## Data availability statement

The original contributions presented in the study are included in the article/supplementary material, further inquiries can be directed to the corresponding author.

## Ethics statement

The study was conducted in accordance with the Declaration of Helsinki, and approved by the Ethics Review Committee of the Shanghai University of Medicine and Health Sciences (no. 2023-hxxm-01-612,401,197,903,300,537) Shanghai China. Informed consent was obtained from all participants in the study.

## Author contributions

SP and PZ: conceptualization and visualization. YaW and XL: methodology. YC: software. SP, YC, JL, YaW, XL, YiW, SG, MP, and PZ: validation, investigation, and supervision. SP, YC, JL, YiW, SG, MP, YaW, and PZ: formal analysis. SP: resources. YaW, XL, and MP: data curation. SP, YC, PZ, and SG: writing—original draft preparation and writing—review and editing. YC and SP: project administration and funding acquisition. All authors contributed to the article and approved the submitted version.

## Funding

This study was funded by the Kashgar Scientific Research Innovation Team Construction Plan, grant number (no. KYTD202106).

## Conflict of interest

The authors declare that the research was conducted in the absence of any commercial or financial relationships that could be construed as a potential conflict of interest.

## Publisher’s note

All claims expressed in this article are solely those of the authors and do not necessarily represent those of their affiliated organizations, or those of the publisher, the editors and the reviewers. Any product that may be evaluated in this article, or claim that may be made by its manufacturer, is not guaranteed or endorsed by the publisher.
